# Circulatory contributors to the phenotype in hereditary hemorrhagic telangiectasia

**DOI:** 10.3389/fgene.2015.00101

**Published:** 2015-04-09

**Authors:** Claire L. Shovlin

**Affiliations:** ^1^ NHLI Cardiovascular Sciences, Imperial Centre for Translational and Experimental Medicine, Imperial College LondonLondon, UK; ^2^ Respiratory Medicine, Hammersmith Hospital, Imperial College Healthcare NHS TrustLondon, UK

**Keywords:** anemia, cardiac output, hypoxia, hemorrhage, iron deficiency, paradoxical emboli, pulmonary hypertension, venous thromboemboli

## Abstract

Hereditary hemorrhagic telangiectasia (HHT) is mechanistically and therapeutically challenging, not only because of the molecular and cellular perturbations that generate vascular abnormalities, but also the modifications to circulatory physiology that result, and are likely to exacerbate vascular injury. First, most HHT patients have visceral arteriovenous malformations (AVMs). Significant visceral AVMs reduce the systemic vascular resistance: supra-normal cardiac outputs are required to maintain arterial blood pressure, and may result in significant pulmonary venous hypertension. Secondly, bleeding from nasal and gastrointestinal telangiectasia leads to iron losses of such magnitude that in most cases, diet is insufficient to meet the ‘hemorrhage adjusted iron requirement.’ Resultant iron deficiency restricts erythropoiesis, leading to anemia and further increases in cardiac output. Low iron levels are also associated with venous and arterial thromboses, elevated Factor VIII, and increased platelet aggregation to circulating 5HT (serotonin). Third, recent data highlight that reduced oxygenation of blood due to pulmonary AVMs results in a graded erythrocytotic response to maintain arterial oxygen content, and higher stroke volumes and/or heart rates to maintain oxygen delivery. Finally, HHT-independent factors such as diet, pregnancy, sepsis, and other intercurrent illnesses also influence vascular structures, hemorrhage, and iron handling in HHT patients. These considerations emphasize the complexity of mechanisms that impact on vascular structures in HHT, and also offer opportunities for targeted therapeutic approaches.

## Introduction

Hereditary hemorrhagic telangiectasia (HHT) results from a single mutation in a causative gene such as *endoglin*, *ACVRL1* (encoding ALK-1), or *SMAD4*. The hallmark of HHT is the presence of arteriovenous malformations (AVMs), and smaller telangiectatic vessels. Additional phenotypic patterns are recognized in smaller numbers of patients (Shovlin, 2010; McDonald et al., 2011).

Hereditary hemorrhagic telangiectasia severity is usually assessed with reference to:

• the presence of vascular abnormalities at particular sites;
• their severity (by anatomic or physiologic measurements);
• hemorrhage, and/or
• organ-specific consequences due to blood bypassing critical capillary beds.

Recent data have begun to illuminate a pattern of marked environmental modification of specific aspects of the HHT phenotype, for example in relation to blood flow, bleeding, and thromboses. Many ‘environmental’ modifiers can be consequences of the HHT phenotype itself.

This mini review focuses on secondary and tertiary consequences of HHT vascular structures. These contribute to the full clinico-pathologic spectrum of HHT, and are relevant to angiogenic, developmental, and injury considerations presented elsewhere in this series.

## Systemic AVMs and Cardiac Output

**FIGURE 1:**
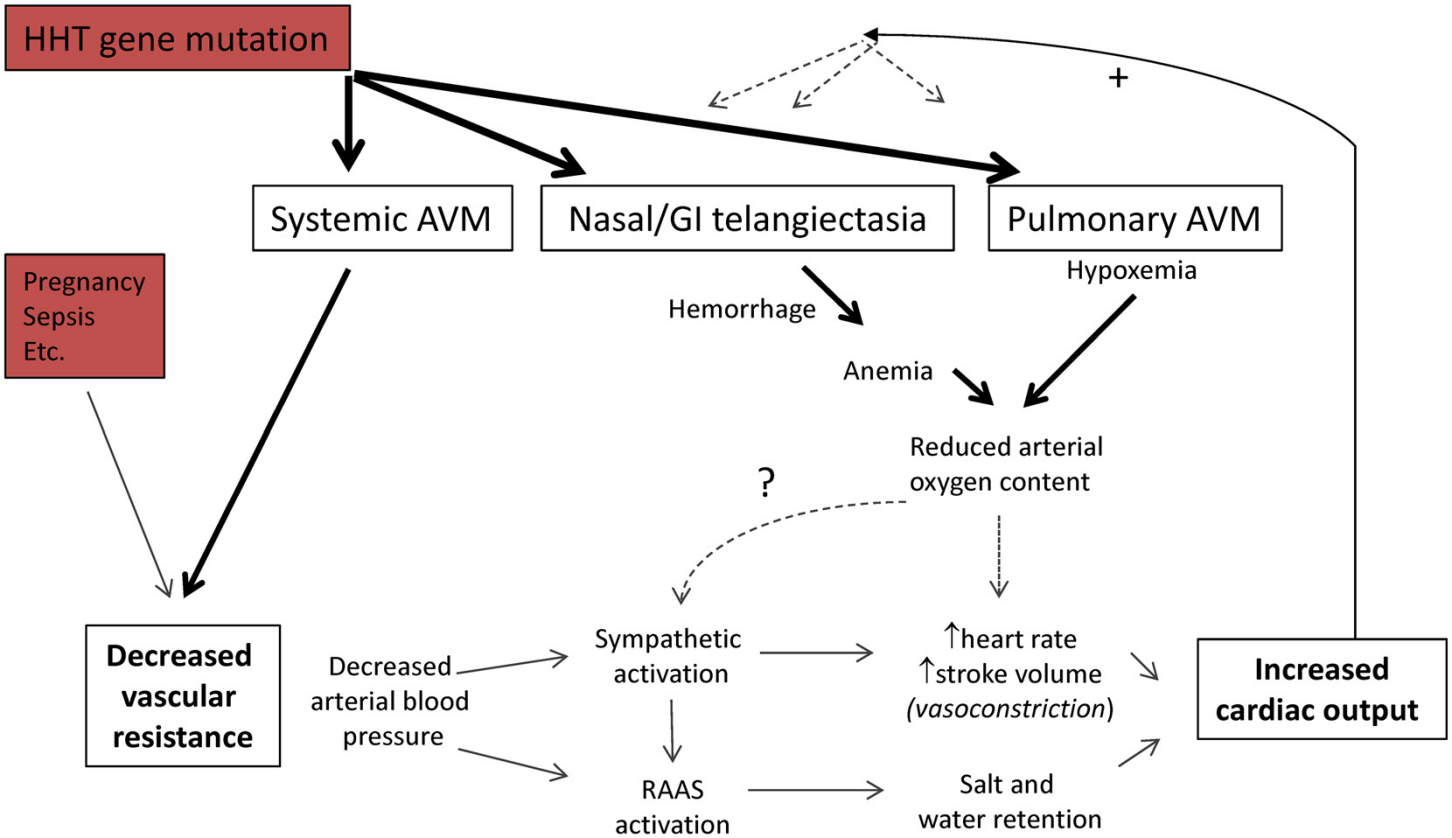
Pathophysiology of high cardiac output states. The cardiac output is the volume of blood ejected each minute by a ventricle, and the product of stroke volume, and heart rate/minute. Systemic vascular resistance is defined by $\frac{mean\,\;arterial\,\;pressure\,-mean\,\;\;\;right\,\;\;atrial\,\;\;pressure}{cardiac\,\;output}$. For further details of clinical states that reduce systemic vascular resistance, see Anand et al., 1993; Anand and Florea, 2001; Mehta and Dubrey, 2009. Abbreviations: RAAS, renin–angiotensin–aldosterone system. Note the potential positive feedback loops influencing HHT vascular structures.

Systemic AVMs are one of the classical pathologies associated with high cardiac output states (Mehta and Dubrey, 2009; **Figure 1**), when cardiac index (cardiac output/body surface area) exceeds 3.9 L/min/m^2^ (Anand and Florea, 2001). Reduced systemic vascular resistance due to the AVMs leads to a fall in arterial blood pressure. Resultant activation of sympathetic and neurohormonal systems increase cardiac output and maintain vital organ perfusion at the expense of salt and water retention (Anand and Florea, 2001; Mehta and Dubrey, 2009). The increases in cardiac output that are needed to preserve arterial blood pressure in the face of severe reductions in systemic vascular resistance may exceed the pump capacity of healthy hearts, leading to high output cardiac failure (Anand and Florea, 2001; Mehta and Dubrey, 2009). High left atrial filling pressures lead to pulmonary venous hypertension (Anand and Florea, 2001; Mehta and Dubrey, 2009).

### Relevance to the HHT Phenotype

**FIGURE 2:**
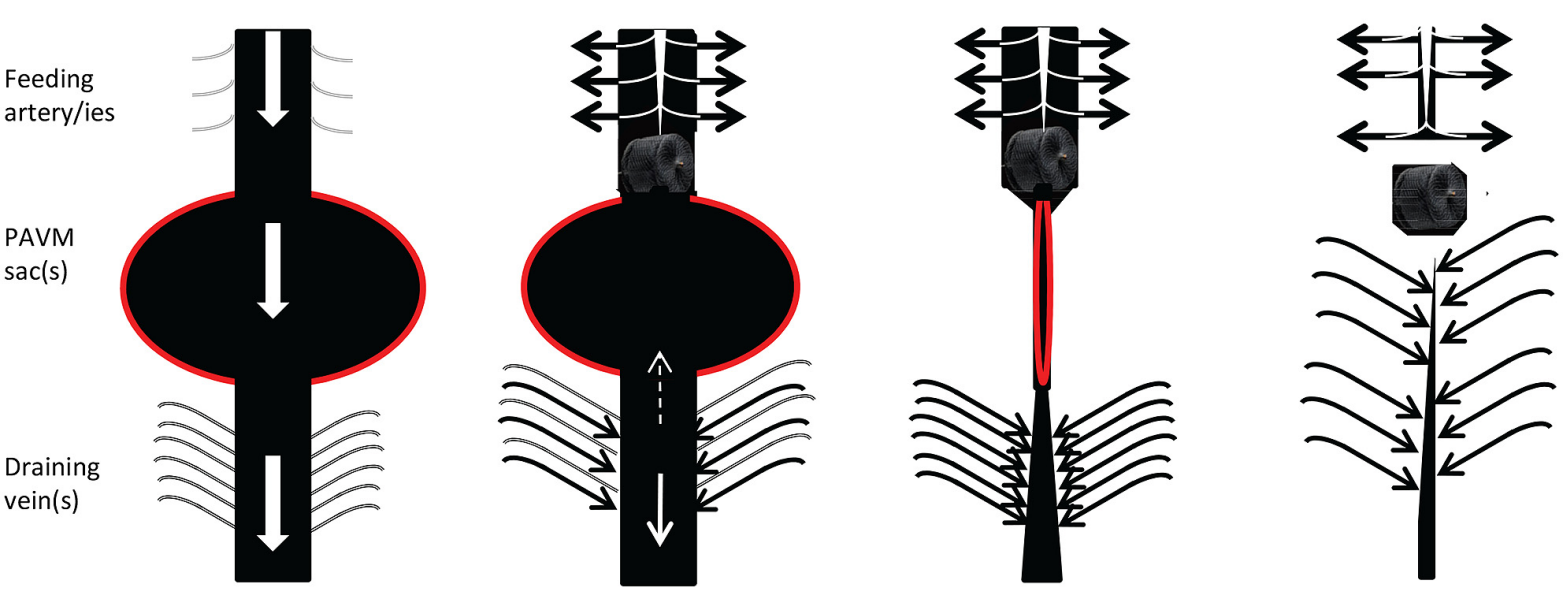
Cartoon of usual behavior of macroscopic, discrete PAVMs pre and post embolization. **(Left)** Preferential blood flow through pulmonary arteriovenous malformation (AVM) sacs (red border) leads to reduced perfusion of non-PAVM associated arteries (gray), dilatation of feeding arteries that commonly appear as second or third order vessels; and dilatation/early filling of draining veins. **Centre left**: immediately following embolization of all feeding arteries, blood flow ceases through the pulmonary AVM, and is redirected to normal arteries. Surprisingly it appears very rare for sac thrombus to embolise before organization. **Centre right and far right**: over subsequent months, assuming feeding arteries remain occluded and the pulmonary AVM does not acquire new feeding arteries, organization, and remodeling leads to regression of the sac, and normalization of diameters of former feeding arteries/draining veins (For patient images, see Howard et al., 2014).

All vessels, including abnormal HHT vascular structures, adapt to the volume and pressure of blood flowing through them. HHT vessels do not behave normally, for example, AVMs do not display the usual adaptation to optimal arterial wall thickness/ lumen radius ratios to minimize wall stress (Owens, 2010). Within specific vascular beds, increased flow modifies vascular structures. In HHT, this is best exemplified by the dilated feeding arteries and draining veins associated with pulmonary AVMs (**Figure 2**). These regress once pulmonary AVMs are embolized and such regression is one of the hallmarks of successful obliteration of the PAVM sacs.

More widely, higher circulating blood volumes are predicted to impact on HHT vascular structures. Multiple HHT series and case reports indicate that in women with HHT, pregnancy can result in the development of new telangiectasia/AVMs, enlargement of existing AVMs, and hemorrhage which may be life-threatening (Ference et al., 1994; Shovlin et al., 1995, 2008; de Gussem et al., 2014).

### Relevance to HHT Clinical Trials

Hemodynamic parameters are increasingly used to evaluate treatment efficacy in HHT clinical trials. Reduction in cardiac index, measured by echocardiography, was the primary efficacy criterion in an evaluation of Bevacizumab in 25 patients with severe hepatic AVMs: 6 months treatment reduced the cardiac index from 5.1 L/min/m^2^ to 4.1 L/min/m^2^, and pulmonary hypertension regressed in five cases (Dupuis-Girod et al., 2012). In a separate study evaluating liver transplantation for severe hepatic AVMs, the mean cardiac index fell from 5.75 L/min/m^2^ to 3.4 L/min/m^2^ (Dupuis-Girod et al., 2010).

## Hemorrhage, Iron Deficiency, and Anemia

Patients with HHT are prone to iron deficiency because of chronic and repeated blood losses from nasal and gastrointestinal telangiectasia. HHT telangiectasia have fragile walls, often lined by a single endothelial layer with no smooth muscle cells or pericytes, despite acting as conduits for blood at arterial pressure (Braverman et al., 1990).

Anemia develops because iron is required to synthesize hemoglobin. Iron handling is generally normal in HHT (Finnamore et al., 2013)- if total body iron stores fall, circulating levels of hepcidin also fall, facilitating iron absorption through the gastrointestinal tract, and iron recycling from hepatocytes and senescent erythrocytes (Ganz, 2013). Over time, if iron intake remains insufficient for needs, body stores are exhausted, leading to iron deficiency. The ‘hemorrhage adjusted iron requirement’ helps predict when iron deficiency is likely to occur, if additional iron supplements are not given (Finnamore et al., 2013).

Additional iron can be ingested orally, and/or administered through iron infusions. However, chronic anemia of a severity sufficient to require red cell (blood) transfusions is common in HHT: One survey reported that 243/915 (26.6%) HHT patients had received a blood transfusion due to epistaxis (nosebleeds, Hoag et al., 2010), and another, that 39/220 (17.7%) patients had required blood transfusions on at least 10 separate occasions (Elphick and Shovlin, 2014).

Limiting HHT blood losses reduces the amount of iron required to avoid iron deficiency and anemia. Strategies include classical otorhinolaryngology surgery; limitation of epistaxis triggers such as hypertension (Purkey et al., 2014), allergic rhinitis/sinusitis (Purkey et al., 2014), dietary (Silva et al., 2013; Elphick and Shovlin, 2014), and drug precipitants (Folz et al., 2005; Devlin et al., 2013; Purkey et al., 2014); and newer medical treatments. Recent attention has focused on hormonal manipulations (Yaniv et al., 2009; Albiñana et al., 2010), thalidomide (Lebrin et al., 2010), tranexamic acid (Gaillard et al., 2014: Geisthoff et al., 2014), and intranasal Bevacizumab (Karnezis and Davidson, 2012; Dupuis-Girod et al., 2014; Riss et al., 2014). There are a number of reports and small, uncontrolled case series reporting the benefits of systemic bevacizumab and other agents, though randomized trials are as yet lacking.

### Anemia, Iron, and Cardiac Output

Chronic anemia leads to reduced systemic vascular resistance and high cardiac outputs in the general population (Porter and Watson, 1953; Anand et al., 1993; Hébert et al., 2004). Treatment of iron deficiency has beneficial effects in general heart failure (Ponikowski et al., 2014): benefits observed in the absence of anemia are attributed to the major iron requirements of tissues with high-energy demands such as the heart and skeletal muscle (Cohen-Solal et al., 2014).

For patients with HHT and hepatic AVMs, the largest prospective series identified iron deficiency anemia as the most common precipitant of high output cardiac failure (Buscarini et al., 2011). In the hepatic AVM-Bevacizumab trial (Dupuis-Girod et al., 2012), the relevance of reduced epistaxis (from 221 to 43 min/month), and modest hemoglobin increase, is not yet known.

### Thromboses in a Hemorrhagic Condition

Despite their frequent hemorrhages, HHT patients are at risk of deep venous thromboses and pulmonary emboli, i.e., venous thromboemboli (VTE; Shovlin et al., 2007; Livesey et al., 2012). A study of 609 consecutive patients with HHT demonstrated an ∼2.5-fold increase in VTE risk with low serum iron (Livesey et al., 2012). The association was evident in “healthy” HHT patients in the community, and appeared to be mediated through elevated coagulation factor VIII (Livesey et al., 2012), which is a well-established risk factor for first and recurrent VTE in the general population (Kyrle et al., 2000; Cosmi et al., 2008; Vormittag et al., 2009; Pabinger et al., 2013; Kovac et al., 2015).

Approximately 50% of HHT patients have pulmonary AVMs and are also at risk of paradoxical embolic strokes because systemic venous blood can bypass the normal pulmonary capillary filter (Shovlin, 2014a; Shovlin et al., 2014). In a study published 15 years ago, 34/67 patients with pulmonary AVMs had evidence of cerebral infarcts on MRI scans (Moussouttas et al., 2000). Myocardial infarcts are also attributed to paradoxical emboli in HHT patients with pulmonary AVMs (Clark et al., 2013). In a series of 497 consecutive patients with CT-proven pulmonary AVMs due to HHT, a low serum iron was a strong risk factor for a clinical ischemic stroke: for the same pulmonary AVMs, the stroke risk would approximately double with serum iron 6 μmol/L compared to mid-normal range (Shovlin et al., 2014). Platelet studies confirmed overlooked data that iron deficiency is associated with exuberant platelet aggregation to 5HT/serotonin (Woods et al., 1977; Shovlin, 2014b; Shovlin et al., 2014).

## Pulmonary AVMs and Hypoxemia

Pulmonary AVMs lead to low oxygen levels in the blood (hypoxemia) because they allow a fraction of pulmonary arterial blood to bypass the pulmonary capillary bed and hence gas exchange (Shovlin, 2014a).

However, in the chronic state, individuals with adequate iron supplies maintain their total arterial oxygen content (CaO_2_) by increasing hemoglobin using an apparently graded erythrocytotic response (Santhirapala et al., 2014a). Similar findings are observed in the general population at altitude, when compensatory responses can be evident within 7 days (Ryan et al., 2014). For pulmonary AVM patients, compensations were shown to be less successful if iron deficiency was present (Santhirapala et al., 2014a). After successful embolization treatment of pulmonary AVMs, hematologic compensatory responses are lost. Patients appeared to reset to the same CaO_2_, unless there was an interim correction of iron deficiency (Santhirapala et al., 2014a).

Pulmonary AVMs also result in high cardiac outputs, with exact mechanisms differing to systemic AVMs, due to the lower blood oxygenation. At rest and on exercise, increased stroke volumes are utilized (Whyte et al., 1993; Howard et al., 2014; Vorselaars et al., 2014). The heart rate normally increases on standing, but in patients with pulmonary AVMs and a sudden drop in SaO_2_ on standing, the heart rate increases further, inversely proportional to the fall in SaO_2_ (Santhirapala et al., 2014b). After correction of hypoxemia by embolization treatments, hemodynamic compensatory responses are lost (Vorselaars et al., 2014).

The loss of both hematologic and hemodynamic compensatory responses following embolization helps explain why exercise capacity, and oxygen consumption are frequently no higher after embolization, despite substantial increases in SaO_2_ (Howard et al., 2014; Shovlin, 2014a; Yasuda et al., 2015).

## HHT-Independent Factors

### Hemodynamics

HHT-independent causes of reduced systemic vascular resistance and high cardiac outputs include exercise, pyrexia, other forms of anemia, pregnancy, sepsis, liver cirrhosis, and use of systemic vasodilating agents (Anand and Florea, 2001; Mehta and Dubrey, 2009).

### Hemorrhage

‘Hemorrhagic’ iron losses occur in multiple settings including menstruation, blood donation, and surgery. All lead to greater iron requirements for the HHT patient (Finnamore et al., 2013). Concurrent coagulation disorders, drugs (Devlin et al., 2013), and even dietary agents (Silva et al., 2013; Elphick and Shovlin, 2014) are reported to increase hemorrhagic losses in HHT.

### Iron Handling

Iron handling is controlled by hepcidin, a liver-synthesized peptide hormone which is normally expressed at lower levels in iron deficiency, facilitating gastrointestinal iron absorption, and iron recycling from macrophages and hepatocytes, through the iron transporter ferroportin (Ganz, 2013). Active bleeding further represses hepcidin expression via erythroferrone (Kautz et al., 2014). However, inflammatory and other chronic disease states often result in inappropriately high hepcidin levels, and failure to absorb oral iron irrespective of doses ingested (Ganz, 2013). In HHT, the hepcidin/ferroportin axis appears to operate normally: generally, hepcidin levels are appropriately low in iron deficiency, displaying similar relationships to ferritin as for healthy controls (Finnamore et al., 2013). However, intercurrent illnesses are predicted to perturb these normal relationships, and aggravate iron deficiency.

### Thromboses

A description of the potential causes of pathological venous and arterial thromboses is beyond the scope of this text. Surprisingly, of 379 patients with HHT who received antiplatelet or anticoagulant therapy (usually to treat or prevent VTE, ischemic strokes or cardiac conditions), 153 (40.4%) reported no change in their nosebleeds, and 9 (2.4%) reported an improvement (Devlin et al., 2013).

Conversely, tamoxifen, raloxifene, tranexamic acid, thalidomide, and Bevacizumab that are used to treat HHT-related bleeding are also recognized in certain circumstances to expose patients to enhanced risk of thrombosis. To date, thrombotic events have not been reported in the HHT clinical trials, but a history of previous thrombosis is considered a contraindication to their use in HHT.

## Conclusion

From a mechanistic perspective, these circulatory contributors to the phenotype in HHT increase the complexity of the disorder. New paradigms are added to current research foci, and new ontology groupings to potential HHT modifier gene lists. Therapeutic targeting of hemorrhage and visceral AVMs in HHT remain major challenges. The states discussed above remind of the potential value of concurrent, simple treatment approaches, particularly preventing or correcting iron deficiency.
